# miR-200c-driven Mesenchymal-To-Epithelial Transition is a Therapeutic Target in Uterine Carcinosarcomas

**DOI:** 10.1038/s41598-017-03972-7

**Published:** 2017-06-15

**Authors:** Jill H. Tseng, Maria Bisogna, Lien N. Hoang, Narciso Olvera, Cristian Rodriguez-Aguayo, Gabriel Lopez-Berestein, Anil K. Sood, Douglas A. Levine, Petar Jelinic

**Affiliations:** 1 Gynecology Service, Department of Surgery, Memorial Sloan Kettering Cancer Center, New York, NY 10065 USA; 2 Department of Pathology, Memorial Sloan Kettering Cancer Center, New York, NY 10065 USA; 3 Gynecologic Oncology, Laura and Isaac Perlmutter Cancer Center, NYU Langone Medical Center, New York, NY 10016 USA; 4 Department of Experimental Therapeutics, Houston, TX 77030 USA; 5 Center for RNA Interference and Non-Coding RNA, Houston, TX 77030 USA; 6 Department of Gynecologic Oncology and Reproductive Medicine, Houston, TX 77030 USA; 7 Department of Cancer Biology, University of Texas, MD Anderson, Houston, TX 77030 USA

## Abstract

Uterine carcinosarcomas (UCSs) are highly aggressive malignancies associated with poor prognoses and limited treatment options. These tumors are hypothesized to develop from the endometrial adenocarcinoma (EAC) through epithelial-mesenchymal transition (EMT). We test this long-standing hypothesis by depleting miR-200, a family of microRNAs critical for EMT, in EAC cell lines. Our data suggest that UCSs do not develop from EACs via EMT. Clinically more relevant, we show that miR-200 expression in UCS cells induces a robust mesenchymal-epithelial transition (MET). Using *in vitro* and murine xenograft models, we demonstrate decreased growth and aggressiveness of miR-200-overexpressing UCS cell lines. Whole transcriptome analysis confirmed changes consistent with an MET and also revealed changes in angiogenic genes expression. Finally, by treatment of UCS-xenografted mice with miR-200c incorporated in DOPC nanoliposomes, we demonstrate anti-tumor activities. These findings suggest that ectopic miR-200 expression using advanced microRNA therapeutics may be a potential treatment approach for patients with UCS.

## Introduction

Uterine carcinosarcomas (UCSs) are rare, highly aggressive malignant neoplasms that account for <5% of all uterine cancers but 16% of uterine cancer-related deaths^1, 2^. These tumors are associated with a poor prognosis and many are diagnosed at an advanced stage. Five-year survival for disease confined to the uterus is 58%, which decreases to 15% for disease extending beyond the uterus^2^. The mainstay of treatment is surgery followed by radiation and/or systemic chemotherapy; however, response to chemotherapy for disseminated disease is approximately 50%^3^.

UCSs are biphasic tumors consisting of both epithelial (carcinomatous) and mesenchymal (sarcomatous) components. The epithelial component is high grade and generally consists of endometrioid or serous histology. The mesenchymal component may resemble histologic components native to the uterus, termed homologous, or harbor components that are not normally found within the uterus, termed heterologous^4–7^. Recent data has shown that UCSs share mutational features similar to serous uterine carcinomas more commonly than endometrioid histologies, have extensive copy number alterations, and almost all harbor somatic *TP53* mutations^8^. UCSs are thought to arise from a monoclonal origin whereby late in tumorigenesis, carcinomatous subclones undergo metaplastic differentiation into sarcomatous cells (conversion theory). This theory is supported by multiple levels of evidence, including the co-expression of cytokeratins and epithelial membrane antigens in carcinomatous and sarcomatous cells^9–11^, as well as concordance of *TP53* and *K-ras* mutations^11–14^, identical patterns of X chromosome inactivation^14–16^, and similar losses of heterozygosity^17^ between carcinomatous and sarcomatous components.

The specific mechanism by which carcinomatous cells undergo metaplastic differentiation has not yet been determined. In accordance with the conversion theory, it is thought that the sarcomatous component is derived from the carcinomatous component through epithelial-mesenchymal transition (EMT)^18, 19^. EMT is a widely studied mechanism leading to cancer progression, metastasis and therapeutic resistance. EMT involves multiple biochemical changes that result in expression of mesenchymal markers, loss of apical-basal polarity and cell-to-cell contacts, cytoskeletal reorganization, morphologic changes from a cobblestone appearance (epithelial) to elongated and spindle-shaped cells (mesenchymal), decreased cellular adhesion, increased migratory capacity and enhanced invasiveness, and increased resistance to apoptosis^20^. This stepwise and dynamic process can lead to full transition of epithelial into mesenchymal cells (complete/full EMT), or partial transition in which cells lose some epithelial characteristics and gain some mesenchymal features (partial EMT)^21^. Additionally, EMT is a reversible process, and mesenchymal-epithelial transition (MET) has been shown to decrease tumor aggressiveness^22, 23^.

miR-200 has been identified as a key element in the EMT pathway^24–26^. This family of microRNAs, consisting of 2 separate gene clusters (chromosome 1: cluster 200b/a/429; chromosome 2: cluster 200c/141), inhibits ZEB1 and ZEB2^27^. ZEB1/2 are transcriptional repressors of E-cadherin, the master regulator of the epithelial phenotype. Decreased E-cadherin expression is an essential event in EMT and is thus considered a hallmark of this process. ZEB1/2, E-cadherin, N-cadherin and vimentin have all been established as core markers of the EMT signature^28, 29^. Compared to endometrial adenocarcinomas (EACs), UCSs have been characterized as having low levels of miR-200 expression associated with a strong EMT signature^18^.

Although UCSs are hypothesized to evolve from EACs^4, 18, 19, 30^, the role of miR-200-driven EMT in the oncogenesis of UCSs has not been previously studied. Furthermore, UCSs are more aggressive than EACs, possibly due to their increased mesenchymal phenotype. miR-200 overexpression can induce MET^22, 23, 31^; however, miR-200-driven MET in UCS has not been previously reported. Here, we test the hypothesis that UCSs arise from a carcinomatous origin *via* miR-200-driven EMT. We show that while EAC cell lines are resistant to full EMT, UCSs readily undergo miR-200-induced MET. We demonstrate that xenografted UCS tumors with miR-200 overexpression show striking changes in morphologic and immunohistochemical phenotype, becoming more epithelial and less mesenchymal-like. Finally, we show that miR-200 overexpression in UCS cells leads to decreased tumor growth and aggressiveness both *in vitro* and *in vivo*. We also explore novel therapeutic approaches to treatment of this aggressive disease.

## Methods

### Cell culture

Ishikawa and MFE-280 EAC cell lines were obtained from Sigma Aldrich; the HEC-251 EAC cell line was obtained from Riken BRC Cell Bank (Japan). Cells were cultured in DME-HG (Ishikawa) or MEM (MFE-280 and HEC-251) supplemented with 10% fetal bovine serum and penicillin/streptomycin (P/S). The JHUCS-1 UCS cell line was obtained from the Riken BRC Cell Bank and maintained in DME:F12 with 15% FBS. SNU-685 cells were obtained from the Korean Cell Line Bank (Korea) and cultured in RPMI 1640 supplemented with 25 mM Hepes, 10% FBS and P/S. Cell lines were maintained under standard conditions. All cell lines were authenticated by the short tandem repeat DNA profiling method (Genetica DNA Laboratories) using the DSMZ database (Leibniz-Insitut DSMZ- Deutsche Sammlung von Mikroorganismen und Zelkukturen GmbH; http://www.dsmz.de/; date of access: May 2015) and tested for *Mycoplasma* by the MSKCC Antibody and Bioresource Core Facility.

### microRNA inhibitor consecutive transient transfection

Ishikawa and MFE-280 cells were consecutively transient transfected with miRNA inhibitors for 10 transfection cycles for 40 days. The cells were seeded to 60–80% confluence in 6-well plates. After 24 hours, the cells were transfected with 10 nM of mirVana miRNA inhibitor (hsa-miR200b3p:MH10492, hsa-miR200c3p:MH11714; Ambion) or negative control #1 (Ambion) using RNAiMax reagent (Invitrogen, Carlsbad, CA, USA). Three days after transfection, the cells were split, seeded and re-transfected as described above. This was repeated every 4 days for a total of at least 40 days (equivalent to 10 transfection cycles). For PCR and immunoblotting assays, cell pellets were collected 3 days after transfection. For functional assays, cells were used when 60–80% confluent (2 days after transfection).

### Generation of miR200c-overexpressing clones

Expression vector pUCori-EF1-Puro-SV40PolyA-Amp^r^ containing a hsa-miR-200c precursor construct (Cell Biolabs) was used to generate stable clones expressing miR-200c. JHUCS-1 and SNU-685 cells were transfected with the miR-200c plasmids using FuGENE HD reagent (Promega) and selected in growth medium containing puromycin at 1 μg/ml. Cell colonies then were individually transferred to 96-well plates, expanded, and maintained under puromycin selection. Quantitative PCR was performed on all clones to confirm miR-200c overexpression.

### RNA extraction and quantitative real-time PCR

Total RNA was isolated from cell pellets and xenograft tumors using the mirVana miRNA isolation kit (Ambion). To quantify levels of miRNA, reverse transcription was performed using the TaqMan® MicroRNA Reverse Transcription Kit, and real-time PCR was performed with TaqMan® miRNA assays (Applied Biosystems). Expression levels were normalized to RNU43. For analysis of mRNA expression, cDNA was synthesized from 2 μg of RNA with the Applied Biosystems™ High-Capacity cDNA Reverse Transcription Kit (Applied Biosystems). PCR was performed on a ViiA™ 7 Real-Time PCR System (Applied Biosystems) using TaqMan® Gene Expression Assays. Expression levels were normalized to GAPDH.

### Immunoblotting

Lysates for immunoblotting were prepared from frozen cell pellets or snap-frozen xenograft tumors using RIPA buffer (Santa Cruz Biotechnology) with 1% Protease Inhibitor Cocktail (Sigma-Aldrich). Protein concentrations were determined using Bradford methodology (Bio-Rad). Proteins (50 μg per load) were resolved onto a 4–20% SDS-PAGE gradient gel (Bio-Rad), transferred onto a nitrocellulose membrane, blocked with 5% milk for 2 hrs, then incubated in primary antibody at 4 °C overnight (ZEB1 H-102, 1:200, sc-25388; ZEB2 H-260, 1:200, sc-48789; E-cadherin H-108, 1:500, sc-7870; N-cadherin, 1:500, ab18203; Vimentin V9, 1:200, sc-6260; β-Actin AC-15 1:10,000, sc-69879). All primary antibodies were obtained from Santa Cruz Biotechnology except for the anti N-cadherin antibody, which was obtained from AbCam. Membranes were then washed in TBST and incubated with horseradish peroxidase-conjugated anti-rabbit or anti-mouse IgG (1:5000, GE Healthcare) for 1 h at room temperature. Blots were developed and visualized using the Amersham™ ECL™ Western Blotting Detection Reagent kit (GE Healthcare Life Sciences). Antibody for β-actin was used as a loading control.

### Cell viability and proliferation assays

Relative cell viability was determined using the VisionBlue™ Quick Cell Viability Fluorometric Assay (BioVision) according to the manufacturer’s protocol. Cells were seeded to 30–40% confluence in 24-well plates. After incubating for the desired amount of time, new media containing 1/10 volume of VisionBlue reagent was added to each well. Cells were incubated at 37 °C for 2 hours. Fluorescence (excitation 530 nm, emission 590 nm) was measured using the Gen5™ Microplate Data Collection and Analysis Software (BioTek).

For the purposes of measuring proliferation, cells were seeded to 30–40% confluence in 6-well plates. To determine proliferation, cells were counted using an automatic cell counter (Bio-Rad). Counts were normalized to the original seeding density. Proliferation for either miR-200 knockdown or miR-200 expressing cells was calculated relative to negative controls.

### Cell adhesion assays

Cell adhesion was measured using the Vybrant™ Cell Adhesion Assay Kit (Molecular Probes) per the manufacturer’s instructions. After trypsinizing, 5 × 10^5^ cells/ml were incubated in fresh media with 5 μM Calcein AM at 37 °C for 30 min. The cells were washed twice with warm media and seeded in 96-well plates. To determine the total number of cells present, baseline fluorescence (excitation 485 nm, excitation 520 nm) was measured immediately after the desired incubation time. Non-adherent cells were then washed away with warm media, followed by PBS. Fluorescence of the remaining adherent cells was measured. Relative adhesion was calculated after normalizing fluorescence of the adherent cells to baseline fluorescence.

### Cell migration and invasion assays

BioCoat™ Matrigel® Invasion chambers and control inserts (BD Biosciences) were used to measure cell invasion and migration, respectively. Cells suspended in serum-free media were added to the upper chamber, and media supplemented with 10% FBS (SNU-685 cells) or 15% FBS (JHUCS-1 cells) was added to the lower chamber. After incubating for 48 hours at 37 °C, cells in the upper chamber that did not migrate or invade were removed with cotton swabs. Using the CytoSelect™ Cell Migration and Invasion Assay Colorimetric Kit (Cell Biolabs), cells that migrated or invaded into the 8 μm pore-size PET membrane were stained with Cell Stain Solution. The inserts were allowed to dry. Cells were then dissociated from the membrane using Extraction Solution. The Extraction Solution containing dissociated cells was transferred to a 96-well plate, and the OD 560 nm was measured using the Gen5 Microplate Data Collection and Analysis Software (BioTek). To calculate relative migration and invasion, the number of cells that migrated or invaded were normalized to the number of cells initially seeded. Fold-change in migration or invasion was calculated by comparing miR-200 knockdown or miR-200 expressing cells to their negative controls.

### TGF-β assays

Ishikawa, HEC-251 and MFE-280 monolayers were incubated with or without human recombinant TGF-β (10 ng/ml; R&D Systems, 240-B-002) for 15 days. Cells were observed for morphologic changes, and pelleted for PCR and immunoblotting assays.

### Murine Xenograft models

Use of animals was overseen by the Research Animal Resource Center under the direction of the Institutional Animal Care and Use Committee at Memorial Sloan Kettering Cancer Center, and was conducted in accordance with all pertinent Federal regulations and policies. The experimental protocol used in these studies was approved by the Institutional Animal Care and Use Committee at Memorial Sloan Kettering Cancer Center. All experiments were performed in accordance with the Institutional Animal Care and Use Committee at Memorial Sloan Kettering Cancer Center guidelines and regulations. We used 5-6 weeks old female mice; strain NOD.CgPrkdcscid IL2rgtm1Wjl/SzJ (Jackson Laboratory; ref. #005557).

JHUCS-1 and SNU-685 cells were grown to 80% confluent monolayers, trypsinized, washed with Phosphate-Buffered Saline, and resuspended 1:1 in Matrigel. Cells (10 × 10^6^) were injected subcutaneously over the posterior flank of mice. To achieve the statistical power of 80% or higher, with a confidence level of 5% (p = 0.05), we used 5 animals per group. Once palpable, the tumor volumes were measured twice weekly. Upon sacrifice of the mice, the tumors were snap-frozen for PCR assays or placed in formalin for immunohistochemistry (IHC).

### Liposomal nanoparticle preparation and *in vivo* administration

1,2-dioleoyl-sn-glycero-3-phosphatidylcholine (DOPC) nanoliposomes were used for miRNA delivery, as previously described^32, 33^. miRNA-DOPC nanoliposomes were prepared using mirVana miRNA mimic negative control #1 (Ambion) or mirVana miR-200c miRNA mimic (has-miR-200c-3p:MC11714; Ambion). One week after JHUCS-1 wild-type cells (5 × 10^6^) were injected subcutaneously over the posterior flank of 5–6 week old NSG mice, the mice were randomly assigned to treatment with control miRNA-DOPC or miR-200c-DOPC. The DOPC nanoliposomes containing miRNA were administered twice weekly *via* tail-vein at a concentration of 200 µg per kg per treatment. Tumor growth was monitored as previously described.

### DNA sequencing

DNA was extracted from frozen cell line pellets (JHUCS-1 and SNU-685) using the DNeasy Blood and Tissue Kit (Qiagen) according to the manufacturer’s instructions. Genomic alterations in 341 key cancer-associated genes were profiled using Memorial Sloan Kettering-Integrated Mutation Profiling of Actionable Cancer Targets (MSK-IMPACT), a custom hybrid capture-based assay for targeted deep sequencing. Captured pools were sequenced on an Illumina HiSeq 2500 platform as 100-base pair paired-end reads, producing at least 250-fold coverage per sample. Data was analyzed to identify single nucleotide variants (SNVs), small indels, and copy number alterations. All candidate mutations were manually reviewed using the Integrative Genomics Viewer^34^.

### RNA-sequencing (RNA-Seq) and analysis

Total RNA was isolated from cell pellets using the mirVana miRNA isolation kit (Ambion). cDNA libraries were prepared using poly-A selection and sequenced on an Illumina HiSeq 2500 platform as 2 × 50-base pair paired-end reads at 30 million reads per sample. Heatmaps for well-established EMT genes were generated using Microsoft® Excel® Version 14.4.9 (Microsoft Corporation, Redmond, WA). Genes with 1.5-fold differential expression (either fold-change ≥1.5 or fold-change ≤0.67) between treated and control groups were analyzed using the Database for Annotation, Visualization and Integrated Discovery (DAVID) bioinformatics resources (DAVID, NIH; https://david.ncifcrf.gov/home.jsp; date of access: February 24^th^ 2016). For Gene Ontology enrichment terms, P-values were calculated using the Benjamini multiple testing correction. P-values < 0.05 were considered statistically significant. For functional annotation clustering, enrichment scores >1.3 were considered equivalent to a P-value of <0.05^35^.

### Immunohistochemistry (IHC)

FFPE sections from each xenografted tumor were stained using antibodies against ZEB1, E-cadherin and vimentin. The IHC conditions for E-cadherin (monoclonal, Ventana) and vimentin (monoclonal, Dako) were previously optimized by the Memorial Sloan Kettering Cancer Center Pathology Core Laboratory. For ZEB1 staining, epitope retrieval was performed using heat by steaming with Citrate buffer (Dako Target Retrieval Solution, #S1699) at pH 6 for 20 minutes. Sections were incubated overnight with the primary antibody at 4 °C (Anti-AREB6, Abcam ab87280, 1:200). Bound antibodies were then detected using Envision + HRP-labeled polymer anti-rabbit for 30 minutes at room temperature (Dako, K4003). For tumors with complete loss of protein expression, staining of blood vessels and stromal cells served as positive internal controls. All slides were reviewed for morphology and IHC staining by an expert gynecologic pathologist.

### Statistics

Results are expressed as mean ± standard deviation (SD). Comparisons of two groups were analyzed using the paired sample T-test or Student’s t-test for independent samples. All calculated P-values were two-tailed, and P-values < 0.05 were considered significant. Error bars depict SD. Statistical analyses were performed using IBM SPSS Statistics for Windows, Version 22.0 (IBM Corporation, Armonk, NY). All assays were done in triplicate and performed 3 times, unless otherwise stated.

## Results

### Characterization of model cell lines

**Figure 1:**
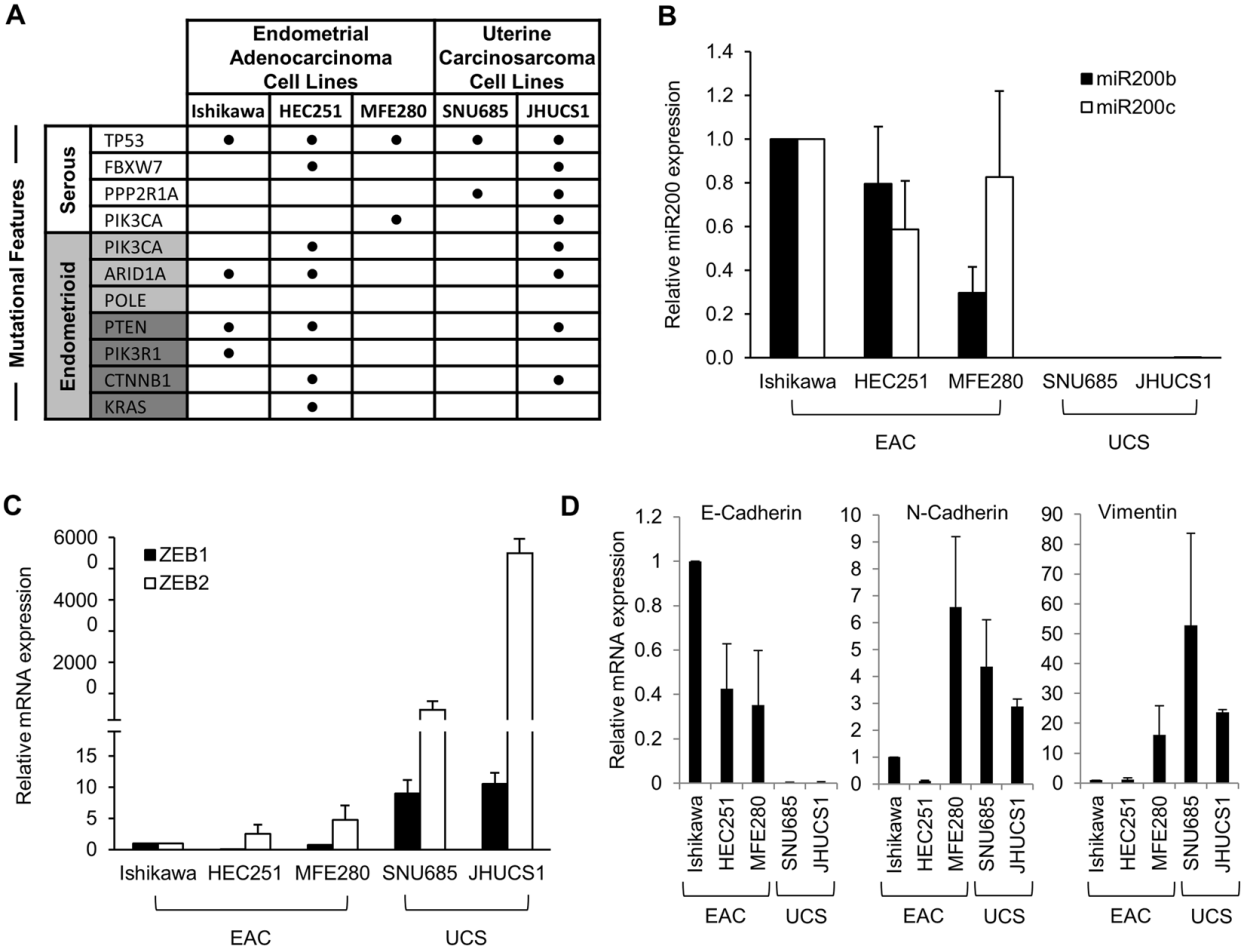
Mutational and gene expression characteristics of EAC and UCS cell lines. (**A**) Mutational landscape of EAC and UCS cell lines. Mutational features (first column) are classified according to endometrial carcinoma histologic subtype: serous (white) or endometrioid (gray). Mutations depicted in dark gray are rarely found in serous carcinomas, while mutations depicted in light gray are more common in endometrioid carcinomas but may also be present in serous carcinomas. (**B**) Relative miR-200, (**C**) ZEB1 and ZEB2, and (**D**) E-cadherin, N-cadherin and vimentin gene expression in EAC and UCS cell lines. Data are normalized to Ishikawa cell line. Data are reported as mean ± SD.

To test the hypothesis that UCSs develop from a carcinomatous phenotype in a miR-200-dependent manner, we selected endometrial adenocarcinoma (EAC) cell lines based on histologic and genetic profiles (Fig. 1A). Mutations more commonly identified in endometrioid adenocarcinomas include *ARID1A* and *POLE*, while those more commonly identified in serous adenocarcinomas include *TP53*, *FBXW7* and *PPP2R1A*. Alterations in *PIK3CA* are frequently found in both endometrioid and serous histologies, whereas *PTEN*, *PIK3R1*, *CTNNB1* and *KRAS* are often mutated in endometrioid but rarely in serous histologies^36–38^. We queried the Broad-Novartis Cancer Cell Line Encyclopedia for endometrioid and serous EAC cell lines. Given that UCSs are proposed to arise from carcinomatous subclones that are predominately p53-mutant, we chose 3 model cell lines with *TP53* mutations: Ishikawa, MFE-280 and HEC-251. Due to the rarity of UCSs, there are a limited number of established UCS cell lines. JHUCS-1s and SNU-685s are both described as having endometrioid carcinomatous components and heterologous sarcomatous components. Neither cell line had previously undergone comprehensive genetic profiling; therefore, we performed MSK-IMPACT testing for further mutational characterization. Mutations consistent with UCSs^8, 39, 40^ were identified in both cell lines. JHUCS-1 cells were found to have an overlapping mutational landscape with mutations in genes seen in both endometrioid and serous adenocarcinomas, including *TP53*, *FBXW7*, *PPP2R1A*, *PIK3CA*, *ARID1A*, *PTEN*, and *CTNNB1*. SNU-685 cells were found to have mutations in *TP53* and *PPP2R1A*, similar to serous adenocarcinomas.

After selecting our cell lines, we sought to examine whether their miR-200 expression and EMT signature profiles were consistent with their named histologic subtypes. We examined the gene expression of miR-200 and several well-established EMT markers (ZEB1, ZEB2, E-cadherin, N-cadherin and vimentin) using TaqMan® Real-Time PCR Assays (Fig. 1B–D). All data were normalized to Ishikawa cells. Compared to the EACs, UCSs had undetectable expression of miR-200b/c (Fig. 1B). In UCS cell lines, we also observed increased expression of ZEB1 and ZEB2, transcription factors upstream in the EMT pathway that are targeted by miR-200 for degradation (Fig. 1C). Finally, increased vimentin and N-cadherin expression and absence of the epithelial marker E-cadherin (Fig. 1D) further confirmed that these cells indeed have features resembling a UCS phenotype. Relative to USCs, EAC cell lines demonstrated decreased levels of ZEB1, ZEB2, N-cadherin and vimentin, and increased levels of E-cadherin. In summary, our model cell lines are representative of EAC and UCS, based on both mutational landscape and miR-200/EMT expression features.

### miR-200-depleted EACs exhibit increased ZEB1 and ZEB2 expression without significant changes in other EMT markers

**Figure 2:**
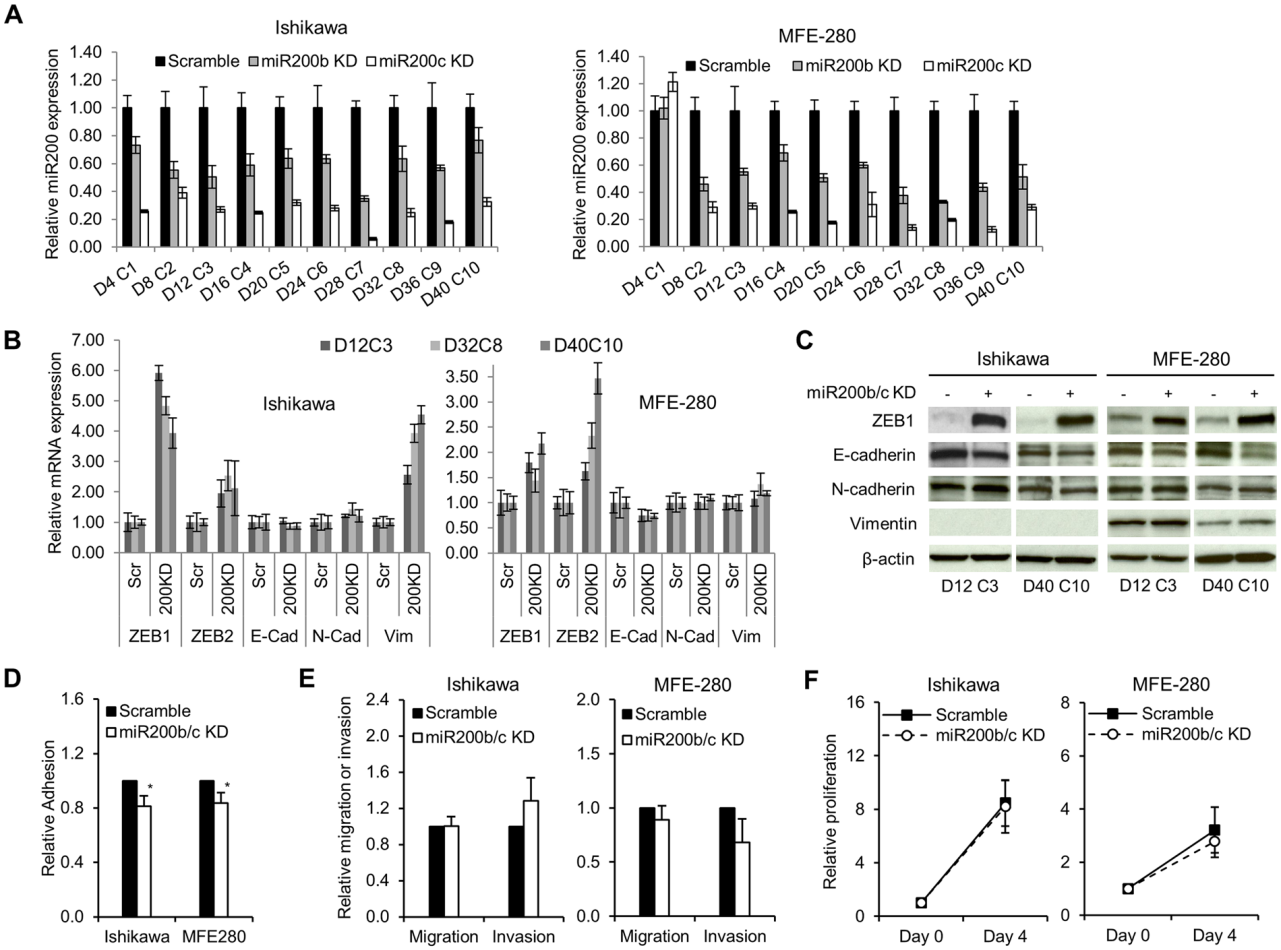
Effects of consecutive transient miR-200 depletion in EAC cell lines. (**A**) Constitutive miR-200b/c depletion in EAC cell lines. Ishikawa and MFE-280 cell lines were transiently transfected with miR-200b/c inhibitor every 4 days for a total of 40 days (D = day, C = transfection cycle). Bar graphs represent relative miR-200b/c expression. Data are normalized to scramble. (**B**) Relative mRNA expression of EMT markers. TaqMan probes for ZEB1, ZEB2, E-cadherin (E-cad), N-cadherin (N-cad) and vimentin (vim) are shown on the bottom. Scramble (Scr) represents control for miR200b/c KD (200KD) samples. Data are normalized to scramble. Representative time points D12C3, D38C8 and D40C10 are shown. (**C**) Protein expression of EMT markers by immunoblotting. Representative early and late time points are shown. Anti-β-actin serves as a loading control. (**D**) Relative adhesion, and (**E**) migration and invasion in miR-200b/c knockdown (miR200b/c KD) vs. scramble-treated cells. (**F**) Relative proliferation measured by cell counts. Data are normalized to Day 0. All graphical data are reported as mean ± SD. *P < 0.05; paired sample t-test. There were no statistically significant differences in migration, invasion or proliferation between miR-200b/c knockdown and scramble-treated cells.

To investigate whether EACs undergo a miR-200-driven EMT resulting in a UCS phenotype, we depleted miR-200b/c in Ishikawa and MFE-280 cells using single-stranded RNAs that inhibit endogenous microRNAs. The cells were transiently transfected with miR-200b/c inhibitors or negative control (scramble) every 4 days. Cyclic transfections were performed for a total of 40 days to allow sufficient time for a complete EMT to take place. Successful miR-200b/c knockdown was confirmed by TaqMan® Real-Time PCR (Fig. 2A). Levels of miR-200b and miR-200c remained decreased over 10 transfection cycles. As expected, constitutive miR-200b/c depletion resulted in increased expression of ZEB1 and ZEB2 in both cell lines by 1.4- to 5.9-fold and 1.9- to 3.5-fold, respectively (Fig. 2B). However, other EMT markers, apart from vimentin in Ishikawa cells, did not show significant changes in mRNA expression.

Analysis of protein expression using immunoblotting reflected these findings, with exception of vimentin in Ishikawa cells (Fig. 2C). Although we detected nearly a 4-fold increase in vimentin mRNA expression, we did not see changes in protein expression in Ishikawa cells. This may be due to a low baseline of vimentin mRNA expression in these cells (delta CT values were at a threshold of detectability), and therefore 4-fold increase in mRNA might not be sufficient to achieve detectable protein expression. Altogether, although ZEB1 levels were robustly increased in both cell lines, other EMT markers were not significantly changed.

Complete EMT results in typical morphologic changes from a characteristic cobblestone shape (epithelial) to an elongated, spindle shape (mesenchymal). We did not observe changes in cellular morphology after miR-200 depletion in either of the EAC cell lines after 40 days of growth (Supplementary Fig. 1), suggesting that the increase in ZEB1/2 expression alone is not sufficient to drive EAC cells to undergo EMT.

### Lack of functional changes in miR-200-depleted EACs further confirms the absence of complete EMT

In addition to loss of cell-cell adhesion and apical-basal polarity, EMT is characterized by cortical actin cytoskeleton reorganization, increased cell contractility and actin stress fiber formation. These events enable directional motility, cellular migration, and invasion. Our data show that, despite miR-200 depletion and subsequent increases in ZEB1/2 expression in EACs, there is no evidence of complete EMT induction. To confirm that these molecular findings reflect the functional behavior of these cells, we assessed cellular proliferation, adhesion, migration and invasion in miR200-depleted compared to control Ishikawa and MFE-280 cells (Fig. 2D–F). For the adhesion assay, cells were labeled with Calcein-AM (CAM) and adhesion was measured 2–6 hours after seeding. Cellular adhesion was decreased by ~16–19% in miR-200b/c-depleted cells compared to control cells of both EAC cell lines (Fig. 2D). For the migration and invasion assays, cells were seeded in Boyden chambers with or without a Matrigel™ Basement Membrane (for measurement of invasion or migration, respectively) and allowed to migrate along an FBS gradient over 48 hours. Cells that successfully migrated or invaded were stained and quantified. Relative to controls, migration and invasion were not significantly different in miR-200b/c-depleted EAC cells (Fig. 2E). To measure cell proliferation, cells were seeded, incubated for 96 hours, and then counted. There was no difference in proliferation between miR-200b/c-depleted EAC cells and control cells (Fig. 2F). In conclusion, although miR-200 depletion and increase in ZEB1/2 expression resulted in a modest decrease in cellular adhesion, there was no evidence of molecular or functional complete EMT.

To explore whether EACs are able to undergo complete EMT by mechanisms other than miR-200 depletion, we treated Ishikawa, HEC-251 and MFE-280 cells with TGF-β, a well-defined and potent inducer of a full EMT response (Supplementary Fig. 2). Changes in miR-200, mRNA and protein expression were assayed 15 days after the treatment. In all cell lines, treatment with TGF-β resulted in elevated levels of ZEB1, ZEB2 and N-cadherin compared to controls; decreased E-cadherin and increased vimentin mRNA expression were modest in MFE-280 cells, but inconsistent in Ishikawa and HEC-251 cells. There were no appreciable differences in cellular morphology in any of the EAC cell lines. Taken together, our data suggest that the only consistent EMT-like changes achievable in EAC cells, either by constitutive miR-200 depletion or exogenous TGF-β treatment, are increases in ZEB1 and ZEB2 expression. However, these changes are not sufficient to modify the molecular, morphologic or functional properties of EAC cells from epithelial- to mesenchymal-like.

### Ectopic miR-200c overexpression in UCSs induces MET

**Figure 3:**
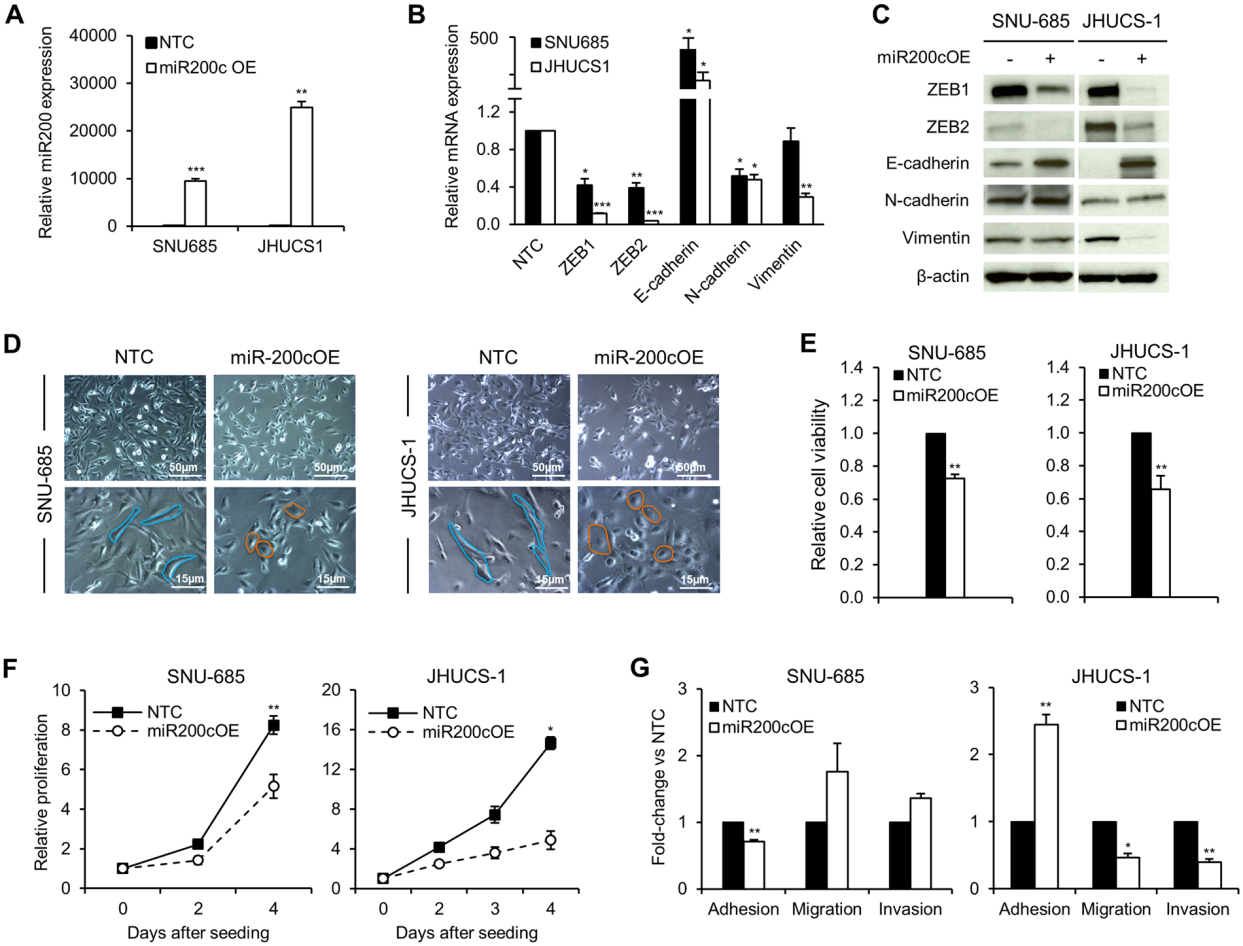
Effects of ectopic miR-200 overexpression in UCS cell lines. (**A**) miR-200c overexpression in SNU-685 and JHUCS-1 cell lines. Bar graphs represent relative miR-200c expression. Data are normalized to non-targeting controls (NTC). (**B**) Relative mRNA expression of EMT markers. miR-200c overexpression data are normalized to NTC. Changes in SNU-685 vimentin mRNA expression were not statistically significant. (**C**) Protein expression of EMT markers by immunoblotting. Anti-β-actin serves as a loading control. (**D**) Morphologic changes in miR-200c-overexpressed UCS cells. Rows represent lower (top) and higher (bottom) magnifications. The spindle-shaped, elongated control cells are outlined in blue. The cobblestone-shaped miR-200c-overexpressed cells are outlined in orange. (**E**) Relative cell viability measured by VisionBlue™ assay 2 days (SNU-685) and 3 days (JHUCS-1) after seeding. Data are normalized to NTC. (**F**) Relative proliferation measured by cell counts. Data are normalized to Day 0. (**G**) Relative adhesion, migration and invasion. There were no statistically significant differences in migration or invasion between miR-200c-overexpressed and control SNU-685 cells. All graphical data are reported as mean ± SD. *P < 0.05, **P ≤ 0.01, ***P ≤ 0.001; paired sample t-test.

Thus far, our data indicate that EACs undergo only partial EMT, and that neither miR-200b/c depletion nor TGF-β treatment is sufficient to induce a full transition from an EAC to a UCS phenotype. Given that EMT is a reversible process, we sought to explore whether miR-200 overexpression in UCS cells would cause MET. While EMT contributes to cancer metastasis and progression, the reverse process, MET, is thought to decrease tumor aggressiveness. We hypothesized that miR-200 overexpression in UCS cells would result in a less aggressive, epithelial-like phenotype. We achieved stable miR-200c overexpression (miR-200c OE) by transfecting JHUCS-1 and SNU-685 UCS cells with an expression vector containing a miR-200c precursor construct and then performing clonal selection. Using TaqMan® RT-PCR, we confirmed 9,457-fold and 24,961-fold increased miR-200c expression in SNU-685 and JHUCS-1 clones, respectively (Fig. 3A). The expression levels of miR200c in miR-200c-overexpressing SNU-685 were comparable to the EAC cell lines, whereas miR-200c-overexpressing JHUCS-1 cells had approximately a 14-fold increased expression when compared to EAC cell lines (Supplementary Fig. 3). Compared to non-targeting controls (NTCs), clones with miR-200c OE demonstrated changes in gene expression consistent with MET (Fig. 3B). E-cadherin levels were significantly increased (412-fold in SNU-685 and 199-fold in JHUCS-1 clones), while ZEB1, ZEB2 and N-cadherin mRNA levels were significantly decreased. Vimentin mRNA levels decreased by 71% in JHUCS-1 cells with miR-200c OE, while no significant changes were observed in SNU-685 cells. Similar alterations in protein expression were seen by immunoblotting (Fig. 3C). Furthermore, miR-200c OE in both cell lines resulted in morphologic changes indicative of MET. As shown in Fig. 3D, control UCS cells were elongated and spindle-shaped, characteristic of a mesenchymal morphology. In comparison, cells with miR-200c OE appeared more cobblestone-shaped, consistent with an epithelial morphology.

Next, we investigated the effects of miR-200c OE on biological processes. We measured cell viability using the VisionBlue™ assay. Compared to NTCs, UCS cells with miR200c OE demonstrated significantly decreased cell viability 48 to 96 hours after seeding (Fig. 3E). These results were orthogonally validated by measuring cell proliferation. UCSs with miR-200 OE exhibited significantly decreased cellular proliferation compared to control cells (Fig. 3F). Four days after seeding, proliferation was decreased by ~37% and ~67% in miR-200 overexpressing SNU-685 and JHUCS-1 cells, respectively. We then examined the effects of miR-200c OE on cellular adhesion, migration and invasion using methodology as previously described. JHUCS-1 cells overexpressing miR-200c demonstrated increased adhesion as well as decreased migration and invasion, suggestive of biological MET changes (Fig. 3G). Unexpectedly, SNU-685 cells with miR2-200c OE showed decreased adhesion and non-statistically significant differences in migration or invasion, compared to SNU-685 NTCs. Altogether, our data show that increased miR-200c is sufficient to evoke molecular and biological changes consistent with MET, fully in JHUCS-1, and partially in SNU-685 cell lines.

### Whole transcriptome sequencing confirms MET changes in miR-200 overexpressed UCS cells and suggests a role for miR-200 in the regulation of angiogenesis

**Figure 4:**
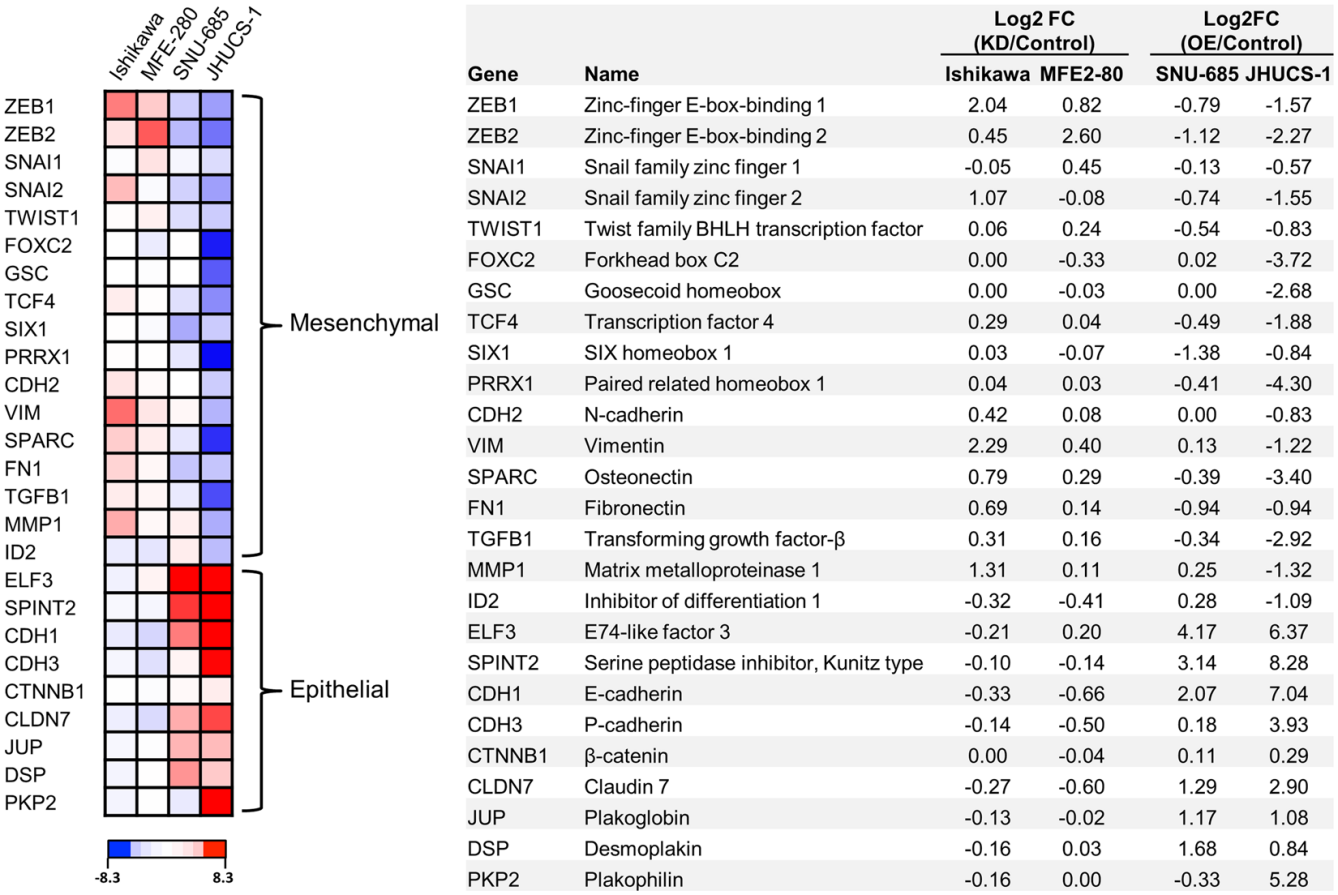
Differential expression of EMT/MET-related genes in EAC-miR-200 depleted and UCS-miR-200 overexpressed cells. (**A**) Heatmap of RNA expression for 26 EMT/MET-related genes. Relative RNA expression of miR-200b/c-depleted vs scramble-treated Ishikawa and MFE-280 cells, and miR-200c-overexpressed vs non-targeting control treated SNU-685 and JHUCS-1 cells, are shown. Increased and decreased expression are depicted in red and blue, respectively. The top 17 genes are known to have increased expression in the mesenchymal phenotype, while the bottom 9 genes are known to have increased expression in the epithelial phenotype. Relative expression is calculated by log2 fold-change (**B**).

To further characterize the molecular effects of miR-200 modulation, we performed whole transcriptome sequencing (RNA-seq) on EAC cell lines with miR200b/c KD and UCS cell lines with miR-200c OE. For each cell line, two miR-200 KD or miR-200 OE samples were compared to one control sample. We generated a heatmap of well-established EMT/MET-related genes (Fig. 4). Similar to the results from our gene expression studies, miR-200b/c KD in Ishikawa and MFE-280 cells resulted in subtle EMT changes overall. However, stable miR-200c OE in JHUCS-1 and SNU-685 cells led to robust upregulation of epithelial genes and downregulation of mesenchymal genes. Additionally, MET changes in the JHUCS-1 cells were more prominent than in the SNU-685 cells. Consistent with our prior findings, this suggests that miR-200c OE drives a more complete MET in JHUCS-1 cells than in SNU-685 cells.

Next, we performed Gene Ontology (GO), KEGG pathway and functional annotation clustering analysis using the DAVID Bioinformatics Resource (DAVID, NIH; https://david.ncifcrf.gov; date of access: February 24^th^ 2016). For this analysis, Ishikawas and MFE-280s were combined into one group, while JHUCS-1s and SNU-685s were combined into another group. Genes with ≥1.5-fold change or ≤0.67 fold change between treatment and control groups were identified, pooled and analyzed. Notably, 913 differentially expressed genes were identified in UCS cells with miR-200c OE, whereas only 185 genes were identified in EAC cells with miR-200b/c KD. In UCS cells that underwent miR-200c OE, GO, KEGG pathway analysis and functional annotation clustering all showed enrichment in MET-related genes (e.g. cellular adhesion, tight/occluding junctions, actin binding/cytoskeleton (Supplementary Figs 4 and 5A). Genes associated with angiogenesis were also enriched, with increased expression of 5/6 anti-angiogenic gene decreased expression of 11/15 pro-angiogenic genes (Supplementary Fig. 5B), suggesting that miR-200-targeted genes may play a role in the inhibition of angiogenesis. DAVID analysis failed to demonstrate any significant enrichment in EAC cells that underwent miR-200b/c KD.

### miR-200c-induced MET in UCS cells leads to decreased *in vivo* tumor growth and epithelial-like morphologic changes

**Figure 5:**
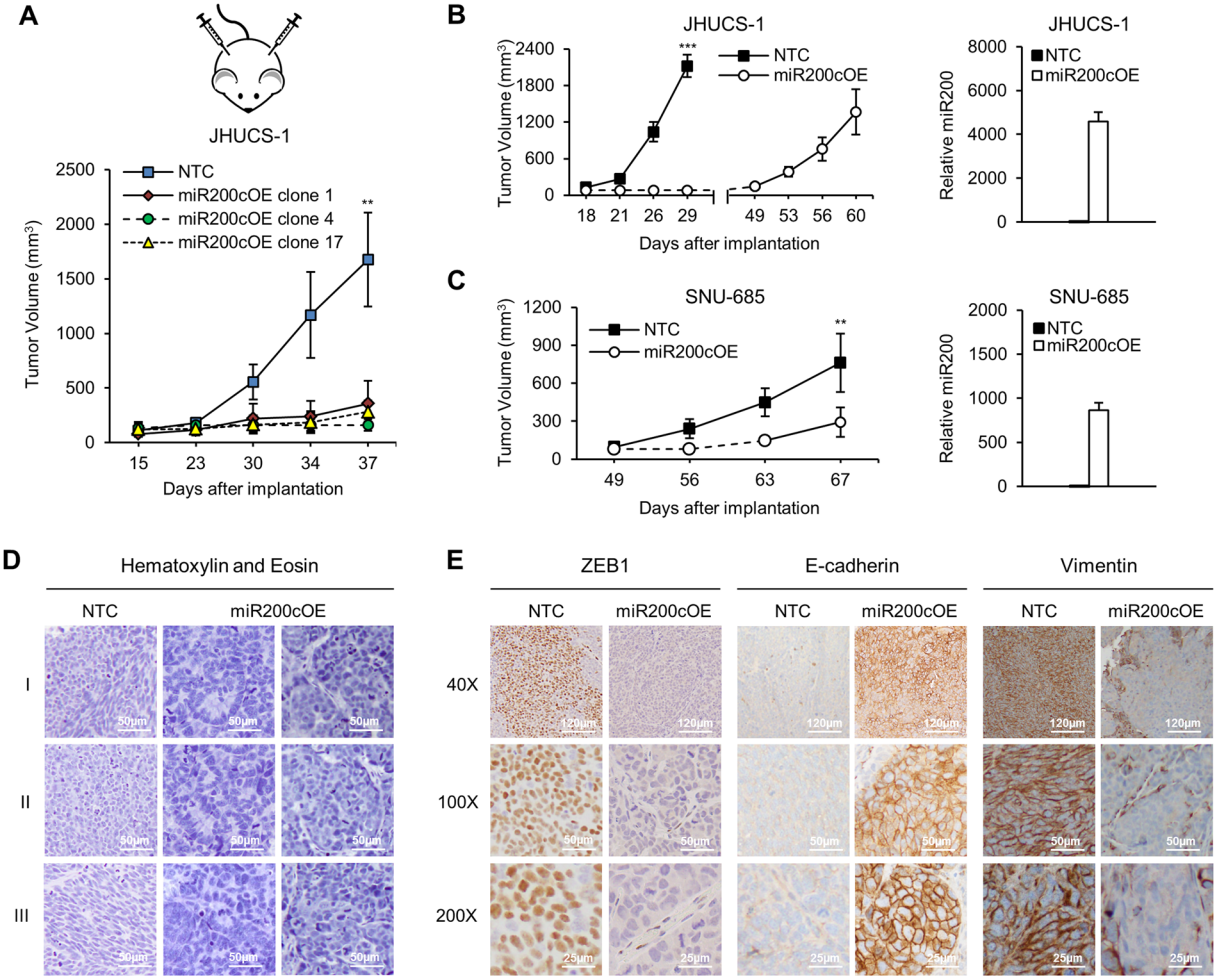
miR-200 overexpression in UCS xenograft tumor models. NSG mice were subcutaneously injected with miR-200c-overexpressed and control cells. (**A**) Tumor growth in control vs. 3 different miR-200c-overexpressed JHUCS-1 clones over 37 days (n = 4 per group). (**B**) JHUCS-1 and (**C**) SNU-685 tumor growth in mice bearing miR-200c-overexpressed vs. control cells over 60 and 67 days, respectively (n = 5 per group). Bar graphs depict relative miR-200c expression in xenografted tumors. All graphical data are reported as mean ± SD. **P ≤ 0.01, ***P ≤ 0.001; Student’s T-test for independent samples. (**D**) H&E staining of JHUCS-1 xenografted tumors. All images were taken at 100X magnification. Scale bar, 50 µm. Rows I, II and III represent different sections of tumor. All NTC were comprised of predominantly spindled cells. (**E**) IHC for ZEB1 (nuclear), E-cadherin (membranous) and vimentin (cytoplasmic). For tumors with complete loss of protein expression, staining of blood vessels and stromal cells served as internal positive controls. Rows represent increasing magnification from top to bottom.

To test whether miR-200c OE leads to decreased tumor growth *in vivo*, we developed murine UCS xenograft models. NTC and miR-200c overexpressing JHUCS-1 cells were injected subcutaneously into NSG mice. Once the tumors were palpable, measurements were performed twice weekly. Similar to our *in vitro* cell proliferation results, mice bearing miR-200c-overexpressed cells had substantially smaller tumors compared to mice bearing control cells (Fig. 5A). To investigate tumor growth over an extended period of time, xenografted JHUCS-1 and SNU-685 tumors were followed for approximately 60 days. Relative to controls, miR-200c OE resulted in increased length of time from injection of cells until formation of measurable tumors (31 days in JHUCS-1; 14 days in SNU-685) and decreased tumor growth (Fig. 5B,C). The effects were more prominent in JHUCS-1 cells versus SNU-685, consistent with our observation that upon miR-200c OE, JHUCS-1 cells exhibit more pronounced biological MET characteristics.

The morphologic changes in JHUCS-1 cells upon stable miR-200c OE further confirm a complete MET (Fig. 5D and E). Xenografted JHUCS-1 tumors were stained with H&E to assess tumor morphology. Histologic sections of control tumors appeared undifferentiated, with no distinct architectural pattern (Fig. 5D). Most cells were small to medium in size and polygonal (Fig. 5D, NTC Row 2); however, some areas contained elongated, spindle-shaped cells akin to a mesenchymal morphology (Fig. 5D, NTC Row 3). In contrast, tumors overexpressing miR-200c showed distinct morphologic changes. The cells were more cuboidal to columnar in shape. They appeared more epithelial, exhibiting cellular polarity, increased cohesiveness, the formation of gland-like structures (Fig. 5D, middle column) and areas of nested architecture (Fig. 5D, right column). We also performed IHC analysis for ZEB1, E-cadherin and vimentin (Fig. 5E). Control tumors showed strong, patchy nuclear staining for ZEB1, a complete lack of staining for E-cadherin, and diffuse, strong cytoplasmic staining for vimentin. In comparison, tumors with miR-200c OE demonstrated significant IHC differences: ZEB1 staining was absent, E-cadherin demonstrated strong, patchy membranous staining, and vimentin staining was minimal. These findings are consistent with our mRNA and protein expression results, and further confirm that JHUCS-1 cells readily undergo complete MET upon miR-200c overexpression.

### Systemic miR-200c-DOPC nanoliposome treatment decreases *in vivo* UCS tumor growth

**Figure 6:**
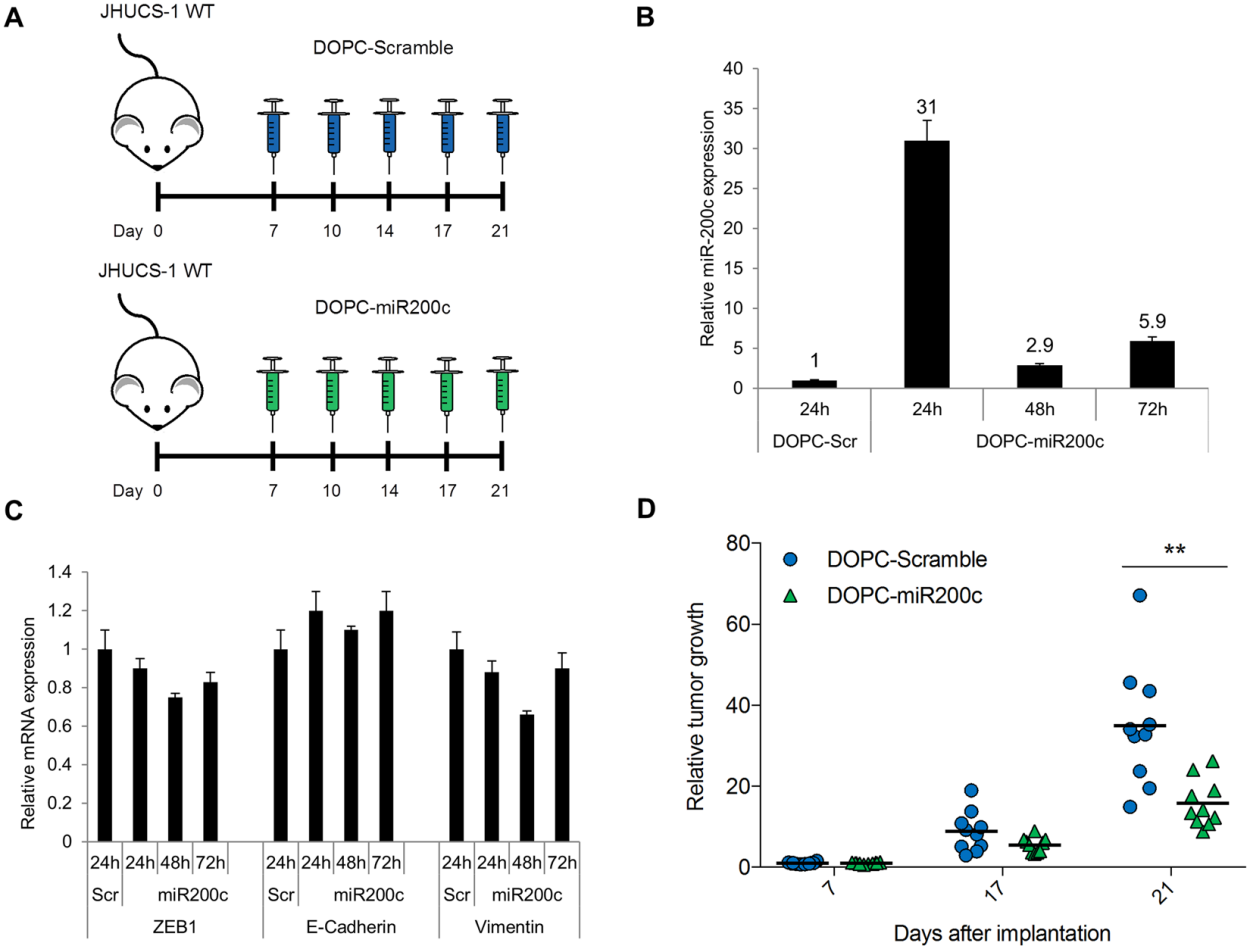
Systemic miR-200c nanoliposome treatment decreases *in vivo* UCS tumor growth. (**A**) NSG mice bearing JHUCS-1 wild-type (WT) UCS tumors were injected with DOPC-Scramble or DOPC-miR-200c nanoliposomes twice weekly by tail vein injection, starting 7 days after subcutaneous cell implantation. (**B**) Relative intra-tumoral miR-200c expression measured by TaqMan RT-PCR. Tumors from mice treated with DOPC-miR200c nanoliposomes were harvested 24, 48 or 72 hours after treatment. Data are normalized to DOPC-Scramble. (**C**) Relative intra-tumoral EMT markers expression measured by TaqMan RT-PCR. Tumor samples were same as in (**B**). TaqMan probes for ZEB1, E-cadherin and Vimentin are shown on the bottom. (**D**) Tumor growth of mice treated with DOPC-Scramble or DOPC-miR200c nanoliposomes (n = 10 per group). Mean tumor size is represented by the black horizontal lines. Data are normalized to the mean Day 7 tumor size, and represented as fold-change in tumor growth. There were no statistically significant differences in tumor growth between DOPC-Scramble and DOPC-miR-200c treated groups on Day 7 or Day 17. **P ≤ 0.01; Student’s t-test for independent samples.

Finally, we sought to test whether *in vivo* on-target effects could be achieved using the well-characterized DOPC nanoliposomal method of systemic miR-200c delivery in a murine xenograft model. Mice bearing subcutaneous JHUCS-1 wild-type cells were treated with DOPC-Scramble or DOPC-miR200c nanoliposomes, twice weekly by tail vein injection, beginning on day 7 after cell implantation (Fig. 6A). Upon analysis of intra-tumoral miR-200c expression by real-time PCR, when compared to control, tumors treated with DOPC-miR200c showed a 31, 2.9 and 5.9-fold increase in miR-200c expression 24, 48 and 72 hours post-injection, respectively (Fig. 6B). Also, the real-time PCR data assessing the EMT markers in these tumors showed subtle decrease in ZEB1 and vimentin, and modest increase in E-cadherin expression (Fig. 6C). After 2 weeks of treatment, mice that received DOPC-miR200c treatment showed a 57% decrease in tumor growth compared to mice that received DOPC-Scramble treatment (Fig. 6D). This suggests that, using the DOPC nanoliposome technology, miR-200c is indeed being delivered to the tumor tissue, and that even moderate increase in miR-200c expression and subtle changes in the EMT markers are sufficient to decrease tumor growth.

## Discussion

UCSs are aggressive, biphasic tumors that have a poor prognosis despite current treatment paradigms with surgery, radiation and chemotherapy. These neoplasms are postulated to arise from less aggressive EACs *via* EMT, although this has not been tested. Our results suggest that while UCSs do not appear to develop from EACs by miR-200-dependent EMT, UCS cell lines readily undergo miR-200-induced MET resulting in decreased tumor growth and aggressiveness.

Recent data has shown that 91% of UCSs harbor *TP53* mutations, and many share mutational features with endometrioid and serous uterine carcinomas^8^. Compared to EACs, UCSs generally exhibit decreased levels of miR-200 and E-cadherin, and increased levels of ZEB1/2, N-cadherin and vimentin^18, 19, 41^. Histology and mutational profiles are essential to the hypothesis that UCSs develop from EACs by divergent evolution (termed the conversion theory). Using mutational profiling and analysis of miRNA and mRNA expression, we confirmed that our model cell lines match miR-200 and EMT gene expression profiles representative of EACs (specifically, endometrioid and serous histologic subtypes) and UCSs.

We tested the hypothesis that UCSs evolve from EACs by EMT in a miR-200-dependent manner. Upon consecutive transient depletion of miR-200b/c in EAC cell lines, we found increased expression of ZEB1/2 and decreased cellular adhesion, as expected in EMT. However, no additional molecular or functional changes consistent with EMT were seen. This suggests that miR-200b/c depletion does not drive EACs into a complete EMT. Failure to induce miR-200-dependent EMT in EAC cell lines due to transient and/or insufficient miR-200 depletion is unlikely for several reasons. Firstly, we used miR-200b and miR-200c inhibitors to ensure knockdown of both miR-200 gene clusters. Secondly, the miR-200 knockdown we achieved was sufficient to increase expression of ZEB1 and ZEB2, which are direct targets of miR-200c. Thirdly, these cells were consecutively depleted of miR-200b/c for a prolonged period of time. Furthermore, even in the presence of exogenous TGF-β, a well-described and potent inducer of EMT^42^, the only consistent EMT-associated changes observed were upregulation of ZEB1 and ZEB2. It is more plausible that the effectors downstream from ZEB1 and ZEB2 in EMT-related pathways prevent EACs from undergoing a complete EMT. Altogether, our results lead us to conclude that UCSs are unlikely to develop from EACs via EMT in a miR-200-dependent and exclusive manner.

Conversely, ectopic expression of miR-200c in UCS cell lines resulted in MET, with acquisition of an epithelial-like phenotype and decreased tumor growth. Molecular changes, signaling mechanisms and microRNA regulation of MET have been described previously^43^; however, MET has not been explored in the context of UCSs and EACs. In our study, miR-200c overexpression in both cell lines led to molecular alterations, morphologic changes, and decreased cell proliferation, suggestive of MET. While miR-200 overexpression in JHUCS-1 cells resulted in MET functional changes (increased cell adhesion and decreased cell migration and invasion), we did not observe the same changes in SNU-685 cells (decreased cell adhesion, no significant change in cell migration or invasion). This is likely due to a lack of significantly decreased vimentin expression in miR-200c-overexpressed compared to control SNU-685 cells. Vimentin is a type III intermediate filament widely expressed in mesenchymal cells. In addition to serving as a cellular scaffold, it plays important roles in cell signaling, cell adhesion, migration and survival^44^. For miR-200c-overexpressed JHUCS-1 cells, decreased vimentin expression and readiness to undergo a complete MET may partly be attributed to the mutational profile of this cell line. JHUCS-1 cells harbor a mutation in *CTNNB1*, a gene that encodes for β-catenin. β-catenin has been proposed to transactivate vimentin in breast cancer cells^45^. Functional β-catenin in *CTNNB1-*non-mutated SNU-685 cells may prevent vimentin downregulation. The presence of other mutations in JHUCS-1 cells, such as *PTEN* and *ARID1A*, might also prime these cells to undergo a complete MET upon miR-200c overexpression.

Xenografting studies further confirmed that miR-200c overexpression in JHUCS-1 cells results in MET. Compared to tumors bearing control cells, those with miR-200c overexpressed cells demonstrated an increased time interval from tumor implantation until formation of measurable tumor, as well as decreased overall tumor growth. These findings are similar to previous work performed in breast cancer cell lines^31^. Striking differences were also seen in the histology and IHC staining of the xenografted tumors. Taken together, our *in vitro* and *in vivo* data strongly support the conclusion that JHUCS-1 cells readily undergo miR-200 driven MET, leading to decreased tumor aggressiveness.

In our study, whole transcriptome sequencing of UCS cells with miR-200c overexpression revealed significant enrichment for genes associated with angiogenesis. In general, these tumors had increased expression of anti-angiogenic genes and decreased expression of pro-angiogenic genes. Angiogenesis is essential for tumor growth and development. A recent study exploring the clinical outcomes related to miR-200 expression found that miR-200 plays an important role in metastasis and angiogenesis^32^. Specifically, ectopic miR-200 expression was shown to decrease metastasis, inhibit angiogenesis, and induce normal vascularization. Although different aspects of angiogenesis (i.e. normal vs. pathologic) are ultimately determined by a complex interplay between pro-angiogenic and anti-angiogenic factors, it appears that miR-200 is associated with inhibition of angiogenesis. This may be one mechanism by which increased miR-200 expression in UCS cells leads to decreased tumor growth and metastasis.

Emerging data has implicated EMT as an important mechanism in chemotherapy resistance^46^. There is also abundant evidence linking miR-200 to treatment sensitivity^47^. Ectopic expression of miR-200 has been shown to increase sensitivity of ovarian, breast and endometrial cancer cells to chemotherapeutic agents^48–51^ and enhance radiosensitivity in lung cancer^52^. These studies all emphasize the therapeutic potential that lies in targeted treatment with miR-200c. Difficulties surrounding microRNA therapy include short half-life, rapid renal and hepatic clearance, and cellular targeting^53^. Many of these barriers are circumvented by encapsulating microRNAs in liposomal nanoparticles. DOPC-nanoliposomes carrying siRNAs against multiple genes were shown to be active in xenograft models of various tumors without any detectable distress or toxicity^54^. DOPC-based delivery approach is now well into clinical development. The most recent example is a phase 1 clinical trial with an EphA2-targeted siRNA incorporated into DOPC nanolipsomes (NCT01591356). Here, we demonstrate that systemic miR-200c DOPC nanoliposome treatment in a murine xenograft UCS model can effectively reach the target tissue, increase intra-tumoral miR-200c expression, and decrease UCS tumor growth.

The treatment of UCS continues to be challenging. Adjuvant therapy is important even for early stage disease; however, a truly efficacious treatment regimen has yet to be discovered. miR-200c decreases tumor growth, and may affect angiogenesis and chemosensitivity. With microRNA therapy emerging as a viable and novel therapeutic approach, miR-200c-targeted treatment may be an attractive option for the treatment of this aggressive disease.

## Electronic supplementary material

Supplementary Figures
