# Supplementary material for: miR-200c-driven Mesenchymal-To-Epithelial Transition is a Therapeutic Target in Uterine Carcinosarcomas
Source: Sci Rep. 2017 Jun 15;7:3614. doi: 10.1038/s41598-017-03972-7 (PMC5472620; doi:10.1038/s41598-017-03972-7)
Supplement: Supplementary file 1 — Supplementary Figures [file 41598_2017_3972_MOESM1_ESM.pdf]

miR-200c-driven Mesenchymal-To-Epithelial Transition is a Therapeutic Target in Uterine Carcinosarcomas

Jill H. Tseng, Maria Bisogna, Lien N. Hoang, Narciso Olvera, Cristian Rodriguez-Aguayo, Gabriel Lopez-Berestein, Anil K. Sood, Douglas A. Levine, Petar Jelinic

Supplementary Figure 1. Morphology of constitutively miR-200-depleted EAC cells.

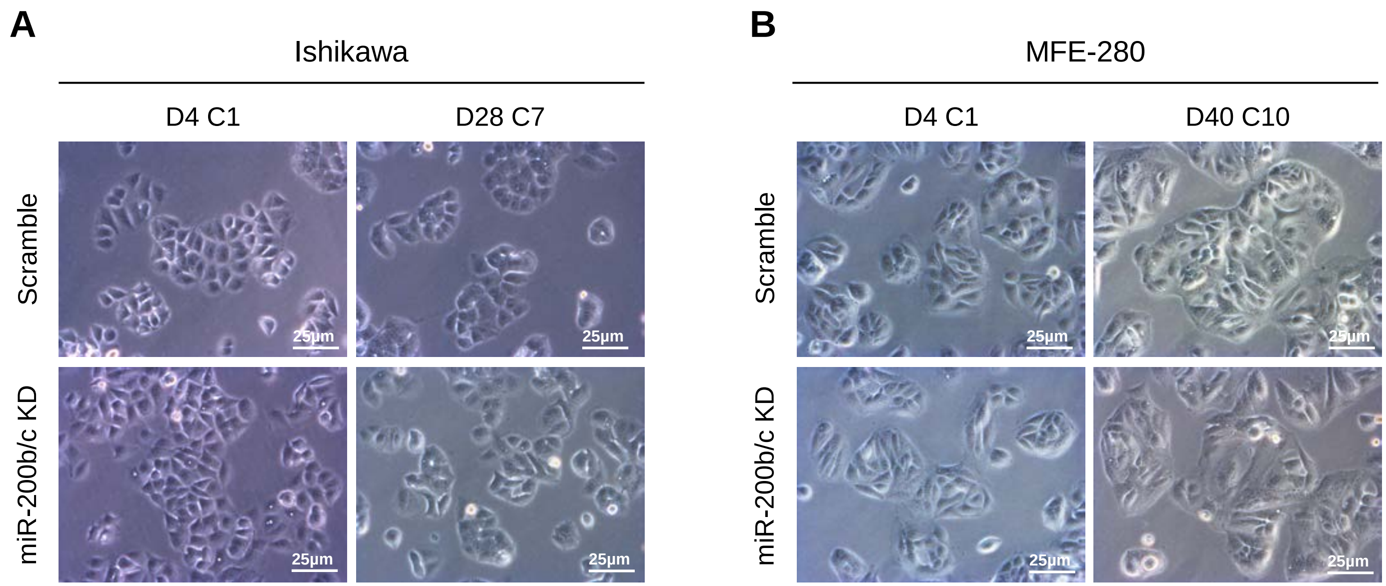

Supplementary Figure 1. Morphology of constitutively miR-200 depleted EAC cells. Appearance of miR-200b/c knockdown vs. scramble-treated (A) Ishikawa and (B) MFE-280 EAC cells. Representative images of select time points are shown (D=day, C=transfection cycle).

Supplementary Figure 2. Exogenous TGF-β treatment of EAC cells.

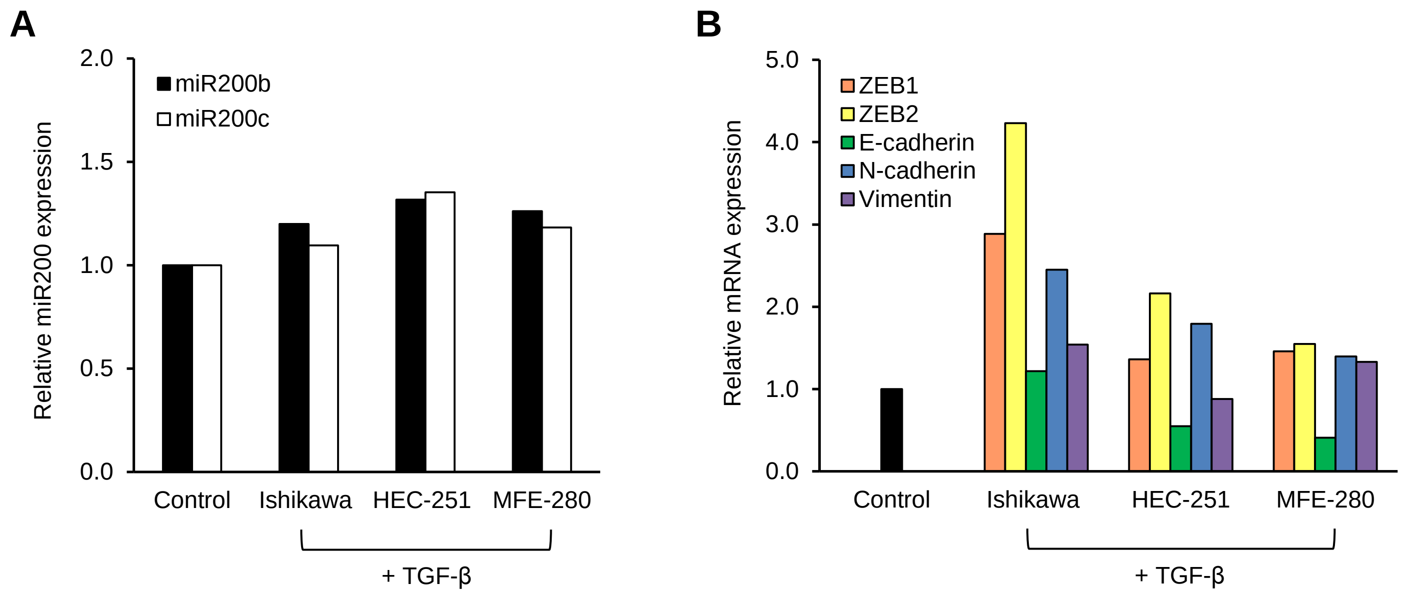

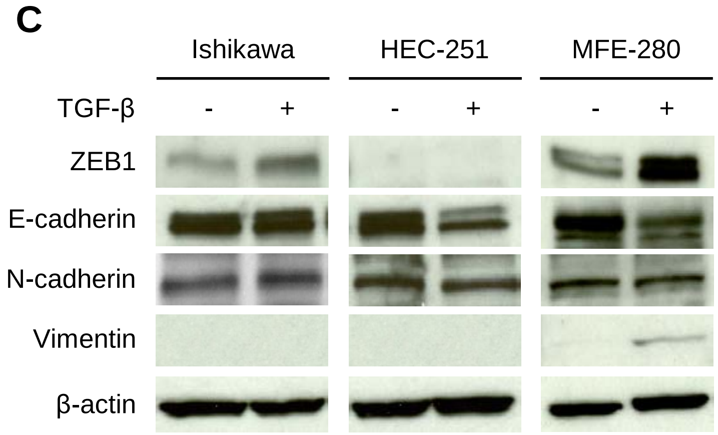

Supplementary Figure 2. Exogenous TGF-β treatment of EAC cells. (A) Relative miR-200 and (B) EMT marker mRNA expression in EAC cell lines treated with TGF-β. Data are normalized to control and are represented as mean values. (C) Protein expression of EMT markers by immunoblotting. Anti-β-actin serves as a loading control.

Supplementary Figure 3. miR200-c expression in EAC and miR200-c-overexpressing UCS cell lines.

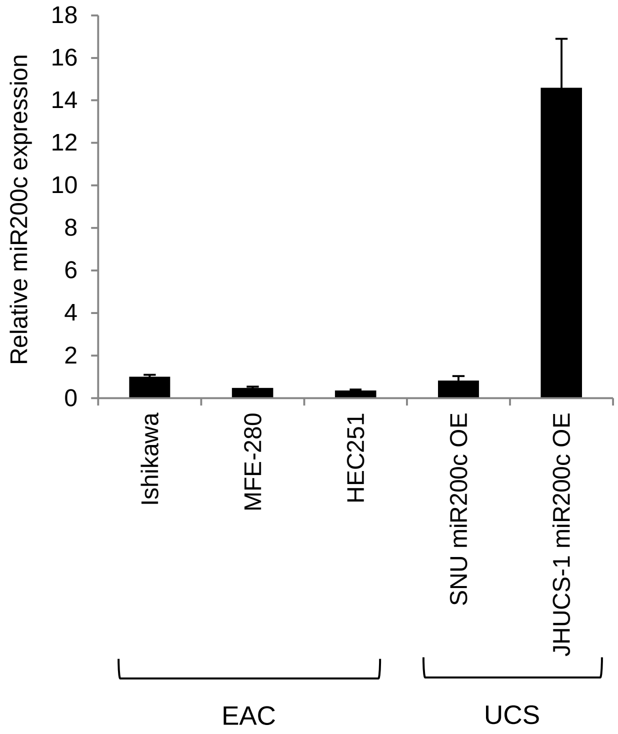

Supplementary Figure 3. Relative miR-200c expression in unmodified endometrial adenocarcinoma (EAC) cell lines compared to miR200c-overexpressing (OE) uterine carcinosarcoma (UCS) cell lines. Data are normalized to Ishikawa cell line. Data are reported as mean ± SD.

Supplementary Figure 4. Whole transcriptome sequencing analysis of UCS cells with ectopic stable miR-200 expression relative to control.

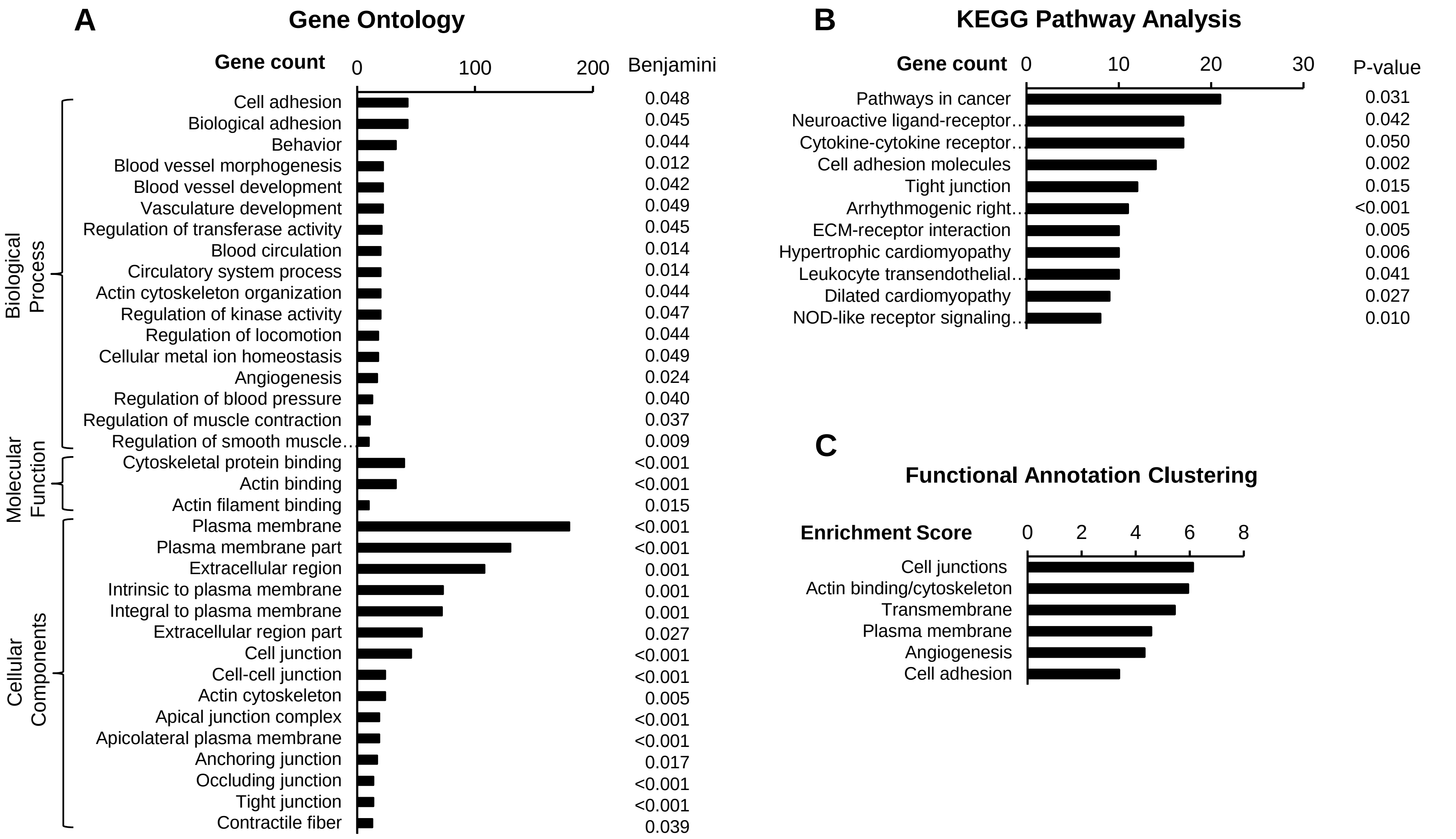

Supplementary Figure 4. Whole transcriptome sequencing analysis of UCS cells with stable ectopic miR-200 expression relative to negative control. Genes with $\geq$ 1.5-fold change or $\leq$ 0.67-fold change between miR-200c-overexpressed and control UCS cells (SNU-685 and JHUCS-1) were analyzed using the DAVID Bioinformatics Resource. (A) Bar graphs depict Gene Ontology, (B) KEGG pathway and (C) functional annotation clustering analysis of significantly enriched terms. For Gene Ontology and KEGG pathway analysis, gene count is represented along the x-axis and enrichment terms are represented along the y-axis. For functional annotation clustering, enrichment score is represented along the x-axis and enrichment clusters are represented along the y-axis. Enrichment scores > 1.3 are equivalent to a P-value of < 0.05.

Supplementary Figure 5. Genes targeted by miR-200c may play a role in the regulation of angiogenesis in UCS.

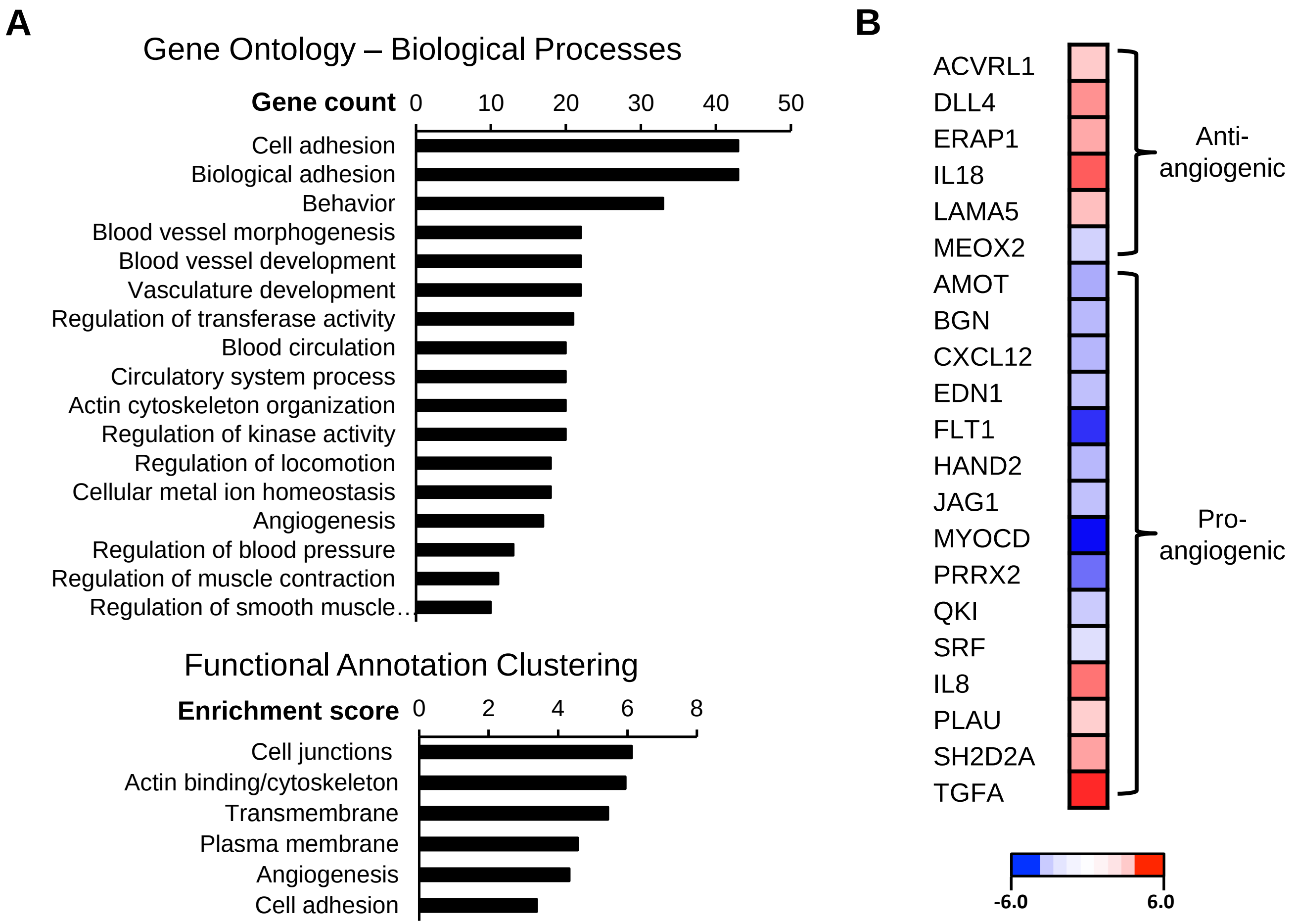

Gene	Log2FC (OE/Control)
ACVRL1	1.24
DLL4	2.59
ERAP1	2.04
IL18	3.85
LAMA5	1.51
MEOX2	-1.08
AMOT	-2.04
BGN	-1.69
CXCL12	-1.78
EDN1	-1.52
FLT1	-5.06
HAND2	-1.72
JAG1	-1.51
MYOCD	-7.09
PRRX2	-3.53
QKI	-1.26
SRF	-0.76
IL8	3.28
PLAU	1.14
SH2D2A	2.20
TGFA	5.06

Supplementary Figure 5. Genes targeted by miR-200c may play a role in the regulation of angiogenesis in UCS. Genes with $\geq$ 1.5-fold change or $\leq$ 0.67 fold-change between miR-200c-overexpressed and control UCS cells (SNU-685 and JHUCS-1) were analyzed using the DAVID Bioinformatics Resource. (A) Bar graphs depict Gene Ontology (top) and functional annotation clustering (bottom) analysis of significantly enriched terms. For Gene Ontology of biological processes, gene count is represented along the x-axis and enrichment terms are represented along the y-axis. P-values with Benjamini correction were calculated for each enrichment term, and all corrected P-values were < 0.05. For functional annotation clustering, enrichment score is represented along the x-axis and enrichment clusters are represented along the y-axis. Enrichment scores > 1.3 are equivalent to a P-value of < 0.05. (B) Heatmap of RNA expression for enriched angiogenesis genes. Increased and decreased expression are depicted in red and blue, respectively. The top 6 genes are anti-angiogenic, while the bottom 15 genes are pro-angiogenic. Relative expression is calculated by log2 fold change.
